# Implementation of an Alzheimer’s Disease Blood Test: Adoption Experience by Memory Care Specialists in a Multi-Center Study

**DOI:** 10.3390/jpm15100469

**Published:** 2025-10-01

**Authors:** Mark Monane, Robert M. Carlile, Kim G. Johnson, Darren R. Gitelman, Lawren A. VandeVrede, Demetrius M. Maraganore, David A. Merrill, Leslie Jacobs, Justine Coppinger, Philip B. Verghese, Tim West, Joel B. Braunstein

**Affiliations:** 1 C2N Diagnostics, LLC, 4340 Duncan Avenue, St. Louis, MO 63110, USA; ljacobs@c2n.com (L.J.); jcoppinger@c2n.com (J.C.); pverghese@c2n.com (P.B.V.); twest@c2n.com (T.W.); jbraun@c2n.com (J.B.B.); 2 Palmetto Primary Care Physicians, Summerville, SC 29486, USA; rmcarlile@palmettoprimarycare.com; 3 Department of Neurology, Duke University School of Medicine, Durham, NC 27710, USA; kim.g.johnson@duke.edu; 4 Advocate Memory Center and the Advocate Aurora Research Institute, Advocate Aurora Health, Downers Grove, IL 60015, USA; 5 Advocate Lutheran General Hospital, Park Ridge, IL 60068, USA; 6 Memory and Aging Center, Weill Institute for Neurology, Department of Neurology, University of California, San Francisco, CA 94158, USA; lawren.vandevrede@ucsf.edu; 7 Department of Neurology, Tulane University School of Medicine, New Orleans, LA 70112, USA; 8 Pacific Neuroscience Institute, Santa Monica, CA 90404, USA; dmerrill@pacificneuro.org

**Keywords:** Alzheimer’s disease, blood biomarker, implementation, personalized medicine, clinical decision-making, memory care specialists, mild cognitive impairment, dementia

## Abstract

**Background/Objectives:** A high-performing blood biomarker (BBM) test for Alzheimer’s disease (AD) represents an accurate, accessible, and scalable tool to aid healthcare professionals (HCPs) evaluating patients presenting with signs or symptoms of mild cognitive impairment (MCI) or dementia. However, implementation of AD blood tests into clinical practice has not been extensively evaluated. The objective of this study was to assess the implementation of the multi-analyte PrecivityAD2™ blood test (C2N Diagnostics, LLC, St. Louis, MO, USA) into the clinical workflow of memory care clinics. **Methods:** A total of 8 HCPs (neurologists, geriatricians, geriatric psychiatrists) who served as site directors from 8 outpatient sites that evaluated 203 cognitively symptomatic patients were included in this sub-study of the real-world QUIP II Study (NCT06025877). Implementation of this blood test was assessed through surveying these HCPs using published frameworks including the Technology Acceptance Model, net promoter score, and forced choice preference questions. These assessments were analyzed using Wilcoxon signed-rank test, Fisher’s Exact test, and Wilcoxon signed-rank test, respectively. **Results:** HCPs reported acceptance scores that averaged 9.6 out of 10 (*p* < 0.0001, effect size 0.840): the test’s contribution to clinical decision-making as well as the ease of understanding test results received the highest ratings. The net promoter score was 75 (*p* < 0.0001), exceeding the typical benchmark of 30 reported as good levels of satisfaction in healthcare settings. The APS2 results and individual blood analyte results were rated with similar preference around their roles in HCP clinical decision-making. **Conclusions:** The results indicate early evidence of user acceptance and recognition by HCPs that this AD blood test can personalize the clinical care pathway for evaluating cognitively symptomatic patients.

## 1. Introduction

The increased use of biomarkers to improve diagnosis, risk stratification, and management strategies serves as a potent enabler of personalized medicine efforts [1]. Blood biomarkers in particular allow for the development of noninvasive, multiomic diagnostic tools that provide a unique opportunity to profoundly affect quality, access, and cost of care. A novel application for personalized medicine lies in the clinical care pathway for cognitive impairment due to Alzheimer’s disease (AD) or other causes of cognitive decline, which has historically presented significant diagnostic challenges for healthcare professionals (HCPs), primarily due to the limitations of traditional assessment methods. The AD misdiagnosis rate among HCPs using current standard of care evaluation procedures, such as magnetic resonance imaging, and routine blood tests labs, such as thyroid stimulating hormone and vitamin B12 levels, is 30–50%, as derived from a Medicare database analysis as well as prospective analysis of primary and secondary care clinics [2,3]. Assessments for brain amyloid, the pathological hallmark of AD, using biomarkers such as amyloid positron emission tomography (PET) scan and cerebrospinal fluid (CSF) analysis [4,5], significantly improve AD diagnosis accuracy, but patients often face barriers related to accessibility and contraindications as well as radiation exposure and invasiveness associated with these tests [6,7,8,9]. The recent development of high-performing blood biomarker (BBM) tests has provided a safe, less resource-intensive, and more accessible alternative for the evaluation of brain amyloid pathology [10,11].

There are several imperatives for the use of personalized medicine tests for AD. While AD is a common cause of cognitive impairment in older individuals, it is not the only one, and early diagnosis of the cause of cognitive decline can help HCPs determine the appropriate next steps in care and management [12]. Furthermore, early diagnosis followed by early intervention with pharmacological and non-pharmacological approaches have shown value in the management of AD [13]. The recent FDA approval and coverage decision by CMS of two disease-modifying agents, lecanemab and donanemab, mandates the pathological confirmation of brain amyloid prior to initiating drug therapy: there is a major unmet need for an accessible and accurate method for such a confirmation that is less costly and less invasive than PET and CSF biomarker testing.

The PrecivityAD2™ blood test (C2N Diagnostics, LLC, St. Louis, MO, USA) is a clinically validated, multi-analyte blood test with incorporation of an algorithm for assessing brain amyloid status in patients with cognitive impairment. The clinical validity (test performance versus reference standard amyloid PET scan) has been previously established [14] and recently replicated in an independent cohort of cognitively symptomatic patients where observed accuracy was 91% (95% CI: 86–94%), sensitivity 90% (95% CI: 83–94%) and specificity 92% (95% CI: 84–96%) [15]. In addition, a prospective clinical practice study (*n* = 1213) showed clinical validity of this blood test’s algorithm in both primary and secondary care using CSF analysis (accuracy 88–92%) as the reference standard [2]. Other AD blood tests have undergone evaluation for clinical validity as well [16,17]. Furthermore, the clinical utility of the PrecivityAD2 blood test was demonstrated in the real-world QUIP II Study (NCT06025877), which showed observed changes in HCPs’ decision-making pre and post blood biomarker testing, including changes in clinician-reported probability of AD, medication prescribing, and the ordering of additional brain amyloid testing [18].

However, while BBM tests are currently available, the integration of these tests by HCPs into the clinical care pathway has not been extensively evaluated. Several implementation science frameworks have been developed and utilized to measure the incorporation of a test or a technology. The Technology Acceptance Model (TAM) is based on understanding the users’ incorporation of new technology into an established pathway. The basic construct is that a test or technology will be adopted if the users believe that the test or technology will enhance their performance and/or do so with less effort to use [19]. In order to add to the evidence base on the integration of an AD blood test by HCPs into the clinical care pathway, the objective of this QUIP II sub-study was to assess the implementation of this AD blood test into the clinical workflow of memory care clinics.

## 2. Materials and Methods

### 2.1. Study Participants and Sites

The participants were 8 memory specialists who served as site directors for the QUIP II Study: 100% (8 out of 8 site directors) completed the study survey. Several inclusion criteria were used in the QUIP II Study. These HCPs were required to actively be involved in patient care practice in the United States and have practices that diagnosed and treated patients with mild cognitive impairment (MCI) or dementia aged 55 years and older. The average patient volume was required to be at least 25 visits per week (all patients seen across practice). All memory care specialists were asked to engage in the study data collection using an online electronic survey. Participating sites included three university-based medical centers and five community-based medical practices across the United States.

### 2.2. Study Design

The current study is a sub-study of the Quality Improvement PrecivityAD2 (QUIP II) Clinician Survey (NCT06025877), a prospective, single arm, outpatient study among patients 55 years and older presenting with signs or symptoms of mild cognitive impairment or dementia. The objective of the QUIP II clinical utility study was to assess clinicians’ patient selection and score interpretation of the test and test result: the results have been previously described [18]. The QUIP II study cohort included 203 patients from November 2023 to May 2024 (Supplementary Table S1).

This implementation study was conducted at the conclusion of the QUIP II Study. The co-primary outcomes of this implementation study were the acceptability, satisfaction, and usefulness of the PrecivityAD2 blood test and its test result as evaluated by HCP surveys. The HCPs completed a six-item, end-of-study, self-administered quantitative survey to assess their experience with and acceptance of the blood test. The survey was designed to evaluate both the technical and practical aspects of implementing this blood test in clinical settings. Each survey was administered through a Health Insurance Portability and Accountability Act (HIPAA) compliant survey system (SurveyMonkey^®^, SurveyMonkey, Inc., San Mateo, CA, USA). Survey responses recorded as free text at the end of each survey included qualitative clinician impressions following blood testing. All survey data were anonymized and not linked to any specific study participant.

The study was reviewed and found to be exempt from institutional review board (IRB) oversight by a national IRB (Advarra, Inc., Columbia, MD, USA). The protocol was submitted to central and local IRBs in alignment with institutional policies. All IRBs granted exemption from oversight.

### 2.3. Study Tools

#### 2.3.1. PrecivityAD2 Blood Test and Test Report

The PrecivityAD2 blood test was intended for use in patients aged 55 and older (currently for use in patients 50 and older) presenting with signs or symptoms of MCI or dementia who are undergoing evaluation for Alzheimer’s disease or other forms of cognitive decline. The test requires a blood draw with the specimen sent to a core laboratory that simultaneously quantifies specific plasma amyloid beta and tau peptide concentrations. The Aβ42/40 Ratio and the percent p-tau217 phosphorylation (100*p-tau217/np-tau217, %p-tau217) are ratios calculated from the absolute concentrations of the peptides. Sample analysis via mass spectrometry provides increased specificity that allows for positive identification of the target analyte. Immunoassays can lack analytical specificity and are therefore prone to false-positive or false-negative results with the potential to impact patient outcomes. The PrecivityAD2 test uses immunoprecipitation liquid chromatography–tandem mass spectrometry (LC-MS/MS) methodology, the instruments and analytical workflow for which have been described previously [14,20,21]. The test has been analytically validated, including the plasma preparation, immunoprecipitation, and LC-MS/MS quantitation of %p-tau217 and Aβ42/40 assays. These assays are precise, accurate, sensitive, linear over a wide analytical range, and free from carryover and interference (Supplementary Tables S2 and S3) [20,21]. The PrecivityAD2 blood test is performed as a service available exclusively through the Clinical Laboratory Improvement Amendments (CLIA) certified laboratory at C2N Diagnostics. Furthermore, C2N Diagnostics received accreditation from the College of American Pathologists (CAP).

The Ratios are combined into a proprietary statistical algorithm to calculate the Amyloid Probability Score 2 (APS2), a numerical value ranging from 0–100, that determines whether a patient is Positive (has high likelihood) or Negative (has low likelihood) for the presence of brain amyloid plaques by amyloid PET scan. The PrecivityAD2 blood test has been clinically validated across two independent cohorts of individuals with cognitive impairment as well as multiple external validations in several orthogonal sets of samples using PET and CSF testing as reference standards [2,14,15].

The patient test report includes the numeric APS2, interpretation of Negative or Positive results, and the Aβ42/40 Ratio and %p-tau217 value: there is no indeterminate APS2 result. The results were returned to the HCPs with a median turnaround time of six business days from the date of specimen receipt by the laboratory.

#### 2.3.2. Technology Acceptance Model

The TAM focuses on two constructs that have been found to significantly influence an individual’s acceptance of (or intention to engage) a technology [19]. Two areas of focus are highlighted: perceived usefulness (PU) and perceived ease of use (PEU). The TAM was originally developed for the field of information technology, but has been extended and applied to healthcare to study the acceptance of new technologies and interventions by users [22,23,24]. In this study, we focused on perceived usefulness of the test in clinical practice and perceived ease of use in routine clinical workflows as judged by HCPs.

#### 2.3.3. Net Promoter Score

The net promoter score (NPS) represents the most prominent measure for evaluation of customer experience metrics. The NPS is used in many fields, including software, retail shopping, and healthcare since 2003 [25]. The single question framework is based on a straightforward metric using one key question to assess customer satisfaction and loyalty: “How likely are you to recommend our company/product/service to a friend or colleague?” In this study, the following research question consistent with NPS methodology in other areas was used: “I would recommend the use of the PrecivityAD2 blood test to my colleagues for evaluating cognitive impairment.” The rating scale ranged from 0 (not at all likely) to 10 (extremely likely). The responses define the raters as promoters (scores 9 and 10), passives (scores 6, 7, 8), and detractors (scores 0–6). Promoters are likely to speak positively, detractors are likely to speak unfavorably, and passives do neither regarding the service under evaluation. The score is calculated by adding the percentage of promoters and subtracting the detractors: the NPS range is −100 to 100 [26,27].

#### 2.3.4. Hierarchical Preference/Constant Sum Methodology Using Coin Analysis

Hierarchical preference assessments generally include presenting a set of choices to the rater and asking them to rank the options in order of preference. Constant-sum methodology takes this assessment one step further by asking the rater to allocate a fixed number of points, for example, across the choices in terms of importance or value. In this study, we asked the participants to assign 10 coins to quantify the relative value of the APS2 results as well as the individual analyte ratio results in clinical decision-making in the evaluation of cognitively symptomatic patients with signs or symptoms of AD [28,29].

### 2.4. Statistical Analysis

For the survey questions on perceived use and perceived ease of use, observed values were compared to a pre-specified benchmark of 5 (consistent with neither agree nor disagree) on a 10-point Likert scale [30] using the Wilcoxon signed-rank test [31]. For the NPS, the observed score was compared to a pre-specified benchmark of 30 (consistent with good HCP satisfaction) using Fisher’s Exact Test [32]. One- tailed *p*-values < 0.05 were considered statistically significant. For the preference analysis, the comparison between APS2 and analyte results was analyzed using Wilcoxon signed-rank test [33]. We measured effect size using Cohen’s d interpretation of effect size [34]. The software version used for this analysis was Python™, version 3.12.7 (scipy 1.31.1, Python Software Foundation, Beaverton, OR, USA).

## 3. Results

### 3.1. Participants

The participants in this study were 8 memory specialists who served as site directors for the QUIP II Study: 100% (8 out of 8 site directors) completed the study survey. Participants were included from three university-based medical centers and five community-based medical practices. Geographic distribution of study sites by US census regions included two sites in the West, three sites in the Midwest, and three sites in the South.

### 3.2. Technology Acceptance Model

With regard to the TAM evaluation, the study of the 8 participants revealed high acceptance rates among HCPs: average acceptance score was 9.6 out of 10 (*p* < 0.0001 versus pre-specified expected score of 5, effect size 0.840 consistent with a large effect size), with a median score of 10 (range, 7–10). These scores are consistent with strong support for the test’s implementation in clinical practice, with HCPs’ particularly emphasizing two key aspects: the test’s contribution to clinical decision-making as well as the ease of understanding test results (Table 1).

### 3.3. Net Promoter Score

Among the 8 participants, the NPS associated with the PrecivityAD2 test use was 75 (derived from 6 promoters and 2 passives over 8 respondents) (95% confidence interval, 43–107). This NPS significantly exceeds the benchmark typically reported in healthcare as good customer satisfaction (*p* < 0.0001 versus pre-specified reference score of 30, effect size 0.420 consistent with a medium effect size) (Table 2).

### 3.4. Preference Analysis of APS2 Results Versus Individual Analytes Results

Among the 8 participants, a review of the relative importance of the test report elements showed that both the APS2 report score and individual analyte ratio results were held in similarly high regard, with 5.3 coins assigned to the APS2 results and 4.7 coins assigned to the individual analytes results (Table 3). There was no statistical difference between these two elements of the test report form (*p* = 0.49).

### 3.5. Qualitative Analysis

In the analysis of abstracted comments associated with the free text input by the participants, the findings provide additional information beyond the more quantitative analysis noted in the TAM and preference analysis results. Many of the comments focused on the similar themes as noted in the TAM, such as perceived ease of use and perceived usefulness of the test and were coded into these categories. In addition, themes raised in the preference analysis as well as comments on the overall effect of the test on clinical decision-making were also noted (Table 4).

## 4. Discussion

Implementation studies aim to narrow or close the divide between scientific evidence and clinical practice. Their value lies in uncovering the mechanisms behind intervention success as well as improving the uptake and execution of new approaches [35,36]. These studies generally produce evidence demonstrating that an intervention is effective (“it works”), supports policy decisions (“it addresses an issue”), and guides feasible implementation (“it can be carried out”) [37]. Furthermore, implementation science seeks to “continue the job” of biomedical research, taking evidence-based clinical innovations and testing strategies to move them into wider practice [38]. This QUIP II sub-study addressed real-world implementation of the PrecivityAD2 blood test, aligning with the recent CEOi BBM Workgroup recommendations on the role of blood tests as confirmatory tests to aid HCPs in the diagnosis of AD [10,11]. In this sub-study, we used published frameworks for evaluating the acceptance of new technologies in healthcare, HCP satisfaction, and usefulness of the PrecivityAD2 blood test and its test result. The sub-study results presented here support the incorporation and implementation of an AD blood test into the clinical care pathway to help HCPs rule in and rule out AD in cognitively symptomatic patients: such an AD blood test can facilitate an early and accurate AD diagnosis that can lead to targeted and personalized non-pharmacological and pharmacological inventions, including the opportunity to identify patients who may be candidates for newer disease-modifying treatments. According to the CEOi BBM Workgroup recommendations, in secondary care, following a comprehensive workup, a positive result from a high-performing blood biomarker test prompts a thorough discussion on the risks and benefits of disease-modifying therapy: no additional testing for brain amyloid confirmation may be needed [10].

Feedback from the HCPs outlined the perceived usefulness and perceived ease of use of this test: the association between the test and the TAM outcomes of interest was highly statistically significant and had a large effect size versus the expected benchmark. The high ratings for ease of understanding test results suggest that the test’s output is well-designed for clinical interpretation and application and is aligned with high perceived ease of use. The TAM is highly relevant and appropriate for evaluating the acceptance and use of a new diagnostic test for cognitive impairment for several reasons, including user acceptance and adoption, adaptability to healthcare context, facilitating implementation, comparative analysis, and customization for target audience. Additionally, the RE-AIM model provides another lens to evaluate the implementation of the PrecivityAD2 blood test [39]: reach (8 memory specialists), effectiveness (published evidence of clinical utility), adoption (203 patients), implementation (surveys of clinicians revealed perceived usefulness and perceived ease of use for evaluating cognitive impalement), and maintenance (long-term follow-up not yet evaluated) [40]. These early findings suggest that the integration of AD blood testing can potentially streamline the diagnostic process for AD, providing a more efficient pathway for patient evaluation.

The NPS associated with this AD blood test was 75, exceeding the typical benchmark of 30 reported as good levels of satisfaction in healthcare settings [27,41]. The use of NPS in healthcare has been noted in several patient and HCP scenarios [42,43]. The results of a recent study focusing on HCP satisfaction assessed the value of online, self-directed CME/CE showed that NPS is a strong indicator of the number of HCP participants who will change practice [44]. To the best of our knowledge, the current study presented here is the first to evaluate a blood-based diagnostic test using the NPS methodology. The high satisfaction rates seen in this study suggest that this AD blood testing can effectively support clinical use in the real-world setting.

HCPs indicated that all components of this test report form were influential in decision-making, with 53% of test value assigned to the APS2 result and 47% of test value assigned to the individual analytes results. The hierarchical preference or constant-sum methodology used in this study allowed for HCPs to communicate their preferences in a structured and quantifiable manner, allowing for more impactful information on the value of the test result and informing future quality improvement efforts [45]. The constant-sum methodology used in this analysis may be useful for assessing factors in decision-making and may provide a complement to other ratings scale [29]. Of note, this methodology is not well studied in healthcare scenarios. Yet the measures of relative rather than absolute preferences as well as the alternative to all-or-nothing decisions may have broad implications in assessing and facilitating healthcare decision-making for HCPs and patients [46,47].

In a follow-up question on insurance coverage elicited from the qualitative comments from the participants, we asked, “If allowed for coverage criteria by commercial payers, would the results of the PrecivityAD2 blood test provide sufficient information for Leqembi^®^ prescribing decisions without the need for PET or CSF biomarker testing?” The results were similar to those observed regarding perceived ease of use and perceived usefulness, with a median score of 10 (range, 7–10). This finding has relevant implications for payer coverage policies and underscores the potential to decrease utilization of expensive PET imaging and invasive CSF testing by HCPs that may be driven more by insurance coverage rules rather than preferred clinical practice.

There are several limitations of the study. The number of participants in the study (8 primary respondents who prescribed and reviewed AD blood tests during the study period) was modest. Yet, given the homogeneity of responses across the eight outpatient sites and variety of medical subspecialties, the results are encouraging for blood test implementation in the evaluation of AD or other causes of cognitive decline. The TAM was originally developed for information technology, yet has become an important model used to identify the factors influencing the adoption of information and technologies in the health system. Similar to the limitation outlined for the TAM, the NPS also originated outside of healthcare as a behavioral metric in the business world measuring customer loyalty and satisfaction, and such interpretation of the NPS in healthcare may be influenced by context and across raters [48]. Furthermore, there are also questions around the TAM and NPS methodology regarding whether one number can be used to describe overall ease of use, usefulness, or satisfaction. However, the well-rounded HCP evaluation used in this study on the implementation of a new test was enhanced not only by the reporting of the combination of TAM and NPS results assessed in the context of multiple quantitative survey methods, but also by the qualitative feedback provided as part of the sub-study protocol. In addition, the hierarchal preference model employed here using coins and a fixed choice structure may have led to oversimplification of complex preferences and has not been fully validated in the healthcare setting [49]. Yet the constant-sum choice experiment as employed in this study design represents one of the most applied preference elicitation method measures: the focus on relative rather than absolute preferences is more aligned with assessing and facilitating healthcare decision-making for HCPs and patients than the all-or-nothing decision-making used on other choice experiments [50].

Our study design evaluated perceived usefulness and perceived ease of use as reported by each clinician at the end of the study. This scenario introduces the potential for clinician recall bias. In addition, we focused on specialty clinics in this study as the test is designed to confirm brain amyloid which has traditionally been done by memory specialists. The introduction and adoption of blood biomarker testing into the primary care setting is the logical next step and may decrease wait times for patients proceeding to the secondary care setting. Furthermore, avoiding social desirability bias in studies including surveys is important and challenging. We addressed this issue by ensuring anonymity and confidentiality of the participant data as described above, using indirect questioning and wording, and including open-ended questions (presented in the qualitative analysis) to go beyond the standardized quantitative scales used in this as part of the study design. Lastly, larger, multicenter evaluations that include cost-effectiveness and patient outcomes have recently been developed, and more are underway to help further establish the role of AD blood tests into the clinical care pathway [51,52,53].

## 5. Conclusions

Given recent guidelines and workshop recommendations highlighting the use of AD blood tests in clinical care, these implementation data provide early evidence of clinical usefulness and ease of use as well as HCP acceptance of an AD blood test. LC-MS/MS method specificity and analytical validity as determined through experiments of precision (imprecision and repeatability), accuracy, analytical sensitivity (limit of blank, limit of detection, limit of quantitation), linearity, carryover and interference demonstrate the robustness of the assay methodology. Additionally, strong overall clinical accuracy has been demonstrated. As healthcare systems continue to seek more effective diagnostic tools for facilitating AD diagnosis in clinical practice, the findings from this study provide valuable insights for the broader adoption of AD blood testing in personalized medicine and clinical practice settings.

## Figures and Tables

**Table 1:** Likert Scale Questions on Perceived Usefulness and Perceived Ease of Use.

Question	Median (IQR)	Range	Measure
“The PrecivityAD2 blood test improves my diagnostic certainty in evaluating patients with cognitive impairment”	10 (1.5)	8–10 *	Perceived Usefulness
“The results of the PrecivityAD2 blood tests significantly contribute to the decision-making process in the evaluation of cognitive impairment.”	10 (0.5)	8–10 *	Perceived Usefulness
“The results of the PrecivityAD2 blood test are straightforward for me to understand”	10 (0.5)	8–10 *	Perceived Ease of Use
“The PrecivityAD2 blood test is easy to incorporate into the diagnostic evaluation of the patients with cognitive impairment.”	10 (0)	7–10 *	Perceived Ease of Use

* *p* < 0.0001 for observed score versus pre-specified Likert score of 5, *n* = 8 respondents.

**Table 2:** Net Promoter Score (NPS) on HCP Satisfaction.

Question—Net Promoter Score and Value of BBM Test to Avoid Further Testing	Median	Range	Measure
“I would recommend the use of the PrecivityAD2 blood test to my colleagues for evaluating cognitive impairment”	10	8–10	Net Promoter Score (75) *

* *p*< 0.0001 for observed NPS versus pre-specified NPS of 30, *n* = 8 respondents.

**Table 3:** Influence on APS2 and Individual Blood Biomarkers on Clinical Decision-Making.

Question—Influence on Contribution of BBM Test Report Results	APS2 Results	Analyte Results	Measure
“What is the influence of the particular components of the test—APS2 results (binary result, 0–100 result) versus analyte results (Ab42/40 result, %p-tau217 result) on clinical decision-making (assessing 10 coins among the 4 choices as a function of value)?”	5.3	4.7	Clinical Decision-Making *

* *p* = 0.49 for comparison of APS2 results versus analytes results, *n* = 8 respondents.

**Table 4:** Qualitative Written Comments on Perceived Ease of Use, Perceived Usefulness, and the Preference Analysis of APS2 versus Individual Analytes.

Implementation Measure	HCP Comments
Perceived Ease of Use	“Having the 0–100 scores along with both a tau and a beta-amyloid related value helps move the discussion forward with patients.” “I have stopped doing CSF evaluations for AD given the confidence of the AD2 test to accurately provide results.”
Perceived Usefulness	“PrecivityAD2 has dramatically impacted my practice. It has shortened the time to diagnosis and greatly impacted treatment and diagnostic decision-making. Being able to have a reliable and simple test has cut back on unnecessary diagnostic testing and medication usage and has streamlined my ability to provide appropriate treatments for patients. It is invaluable.” “This test has been a “game changer” in my practice. I’ve done CSF studies for years for AD biomarkers, and thought I would continue to do so. But the AD2 test has influenced my thinking so much that I can reliably use this information to make clinical decisions without the test. I have experience with a variety of treatments for AD, from therapeutic lifestyle medicine (it works), to monoclonal antibodies targeting amyloid, and other investigational drugs through clinical trials, and off-label treatments. I could not do what I do for my patients without this test.”
Preference Analysis of APS2 versus Individual Analytes Results	“Given the high correlation between APS2 and amyloid PET status, it makes sense that the blood test be covered for use instead of more expensive, more risky, and less available PET and/or CSF tests.” “The Precivity test not only gives the absolute values for p-tau217 and amyloid beta ratio, but also the percentage score which is extremely helpful in determining likelihood of AD. Only diagnostic uncertainty is when the amyloid score is near the cutoff, abeta 42/40 ratio is normal but p-tau 217 is high. I do think repeat testing with CSF biomarkers is necessary in these cases.”

## Data Availability

Investigators may request access to anonymized individual patient data and redacted trial documents including raw datasets, analysis-ready datasets, trial protocols, annotated case report form, statistical analysis plan, dataset specifications, and clinical trial report 20 months after trial is complete. Prior to use of the data, proposals need to be approved, and a signed data sharing agreement will then be put in place. All documents are for a predetermined time, typically 12 months.

## Supplementary Materials

The following supporting information can be downloaded at: https://www.mdpi.com/article/10.3390/jpm15100469/s1, Table S1: QUIP II Study Description; Table S2: Analytical Background and Quantification of the PrecivityAD2 Blood Test; Table S3: Analytical Validation Data and Specifications of the LC-MS/MS Assays Used in the PrecivityAD2 Blood Test.
