# Supplementary material for: Implementation of an Alzheimer’s Disease Blood Test: Adoption Experience by Memory Care Specialists in a Multi-Center Study
Source: J Pers Med. 2025 Oct 1;15(10):469. doi: 10.3390/jpm15100469 (PMC12565532; doi:10.3390/jpm15100469)
Supplement: Supplementary file 1 [file jpm-15-00469-s001.zip › jpm-3801339-supplementary.pdf]

Table S1. QUIP II Study Description [18]

Section	Description
Objective	The Quality Improvement PrecivityAD2 (QUIP II) Clinician Survey (NCT06025877) was a prospective, single arm, multi-site outpatient study among patients 55 years and older presenting with signs or symptoms of mild cognitive impairment or dementia. The objective of this study was to assess clinical decision-making associated with the use of a multi-analyte blood biomarker (BBM) test among patients presenting with signs or symptoms of mild cognitive impairment or dementia.
Methods	The memory specialists received education and training on the intended use of this blood test as well as the APS2 result. EDTA blood specimens were collected from participating study subjects, centrifuged, and plasma aliquoted prior to shipping. Samples were shipped on the day of collection using a refrigerated shipping solution to the C2N CAP (College of American Pathologists)-accredited, CLIA (Clinical Laboratory Improvements Amendments)-certified laboratory for analysis. Test results were returned to the ordering study clinician by dedicated fax line.<br><br>The assessment of clinical utility included an evaluation of patient selection and score interpretation by clinicians of the BBM test and its result, the Amyloid Probability Score 2 (APS2). The APS2 result was reported on a scale of 0-100: Negative (APS2 0-47) and Positive (APS2 48-100) results represent low and high likelihood, respectively, for the presence of brain amyloid plaques on amyloid PET scan. After receiving the BBM test results, each clinician completed a survey built within a HIPAA (Health Insurance Portability and Accountability Act) compliant survey system (SurveyMonkey®, SurveyMonkey, Inc., San Mateo, CA). The survey collected patient demographics, clinician information, and feedback concerning pre- and post-BBM test diagnostic certainty, and pre- and post-BBM test patient management plans including medication prescribing and additional brain amyloid evaluation. APS2 results were interpreted by clinicians at their own discretion.<br><br>The primary outcome of the study had two parts: patient selection and score interpretation. Patient selection was evaluated in terms of concordance of clinicians' test ordering with the intended use criteria of the PrecivityAD2 blood test. Clinical decision-making, a measurable proxy for interpretation of the test's APS2 result, was evaluated in terms of changes in clinician-reported probability of AD (0-100%) pre- and post-BBM testing as well as AD drug therapy (acetylcholinesterase inhibitors, memantine, and lecanemab) and additional amyloid brain evaluation pre- and post-BBM testing as reported on the clinician survey.
Results	Concordance with intended use of the BBM test was 99% (200/203). Reasons for non-concordance were test use outside of the intended use, including patients below the age of 55 (n=1) and patients without symptoms of MCI or dementia (n=2). The composite primary endpoint, defined as a change in AD diagnostic certainty, drug therapy, or additional brain amyloid evaluation pre- and post-BBM testing, was 75% ($p < 0.0001$ versus pre-specified threshold of 20% clinically meaningful change).<br><br>Secondary outcomes included the results of each subpart of the primary outcome. Concordance with age and concordance with symptoms criteria were evaluated. Changes in clinician-reported AD diagnostic certainty were measured pre- and post-BBM testing, Changes in medication prescribing as well as additional test ordering were measured pre- and post-BBM testing. Anti-AD medication orders decreased among Negative APS2 patients and increased among Positive APS2 patients ($p < 0.0001$). Additional brain amyloid testing decreased among Negative APS2 patients ($p < 0.0001$).

Reference [18]: 18. Monane, M.; Maraganore, D.M.; Carlile, R.M.; Johnson, K.G.; Merrill, D.A.; Gitelman, D.R.; Sharlin, K.S.; VandeVrede, L.A.; George, K.K.; Wang, J.; et al. Clinical Utility of an Alzheimer's Disease

Blood Test Among Cognitively Impaired Patients: Results from the Quality Improvement PrecivityAD2 (QUIP II) Clinician Survey Study. Diagnostics 2025, 15, 167. https://doi.org/10.3390/diagnostics15020167.

Table S2: Analytical Background and Quantification of the PrecivityAD2 Blood Test [14,20,21]

Section	Description
Step 1	Prior to immunoprecipitation, a known amount of internal standard proteins (ISTDs) is added to all samples, controls, and calibrators to normalize the endogenous peptide concentrations. These ISTDs serve as control measurements throughout the analytical process to increase the precision and accuracy of analyte quantification.
Step 2	The samples undergo immunoprecipitation as well as extraction, separation, and quantification of the analytic peptides by LC-MS/MS. The PrecivityAD2™ test consists of two LC-MS/MS tests from plasma: the Aβ42 and Aβ40 LC-MS/MS assay and the p-tau217 and np-tau217 assay. This process includes samples analyzed in 96 well plates, inclusive of calibration samples and quality control samples.
Step 3	The concentration of the Aβ42 and Aβ40, and p-tau217 and np-tau217 analytic peptides measured by LC-MS/MS are calculated into respective ratios. A proprietary algorithm generates the APS2 score of 0-100.

References: [14] Meyer, M.R.; Kirmess, K.M.; Eastwood, S.; Wente - Roth, T.L.; Irvin, F.; Holubasch, M.S.; Venkatesh, V.; Fogelman, I.; Monane, M.; Hanna, L.; et al. Clinical validation of the PrecivityAD2 blood test: A mass spectrometry-based test with algorithm com-bining %p-tau217 and Aβ42/40 ratio to identify presence of brain amyloid. Alzheimer Dement. 2024, 20, 3179–3192. https://doi.org/10.1002/alz.13764. [20] Eastwood, S.M.; Meyer, M.R.; Kirmess, K.M.; Wente-Roth, T.L.; Irvin, F.; Holubasch, M.S.; Verghese, P.B.; West, T.; Braunstein, J.B.; Yarasheski, K.E.; et al. PrecivityAD2™ Blood Test: Analytical Validation of an LC-MS/MS Assay for Quantifying Plasma Phospho-tau217 and Non-Phospho-tau217 Peptide Concentrations That Are Used with Plasma Amyloid-β42/40 in a Multi-analyte Assay with Algorithmic Analysis for Detecting Brain Amyloid Pathology. Diagnostics 2024, 14, 1739. https://doi.org/10.3390/diagnostics14161739. [21] Kirmess, K.M.; Meyer, M.R.; Holubasch, M.S.; Knapik, S.S.; Hu, Y.; Jackson, E.N.; Harpstrite, S.E.; Verghese, P.B.; West, T.; Fogelman, I.; et al. The PrecivityAD™ test: Accurate and reliable LC-MS/MS assays for quantifying plasma amyloid beta 40 and 42 and apolipoprotein E proteotype for the assessment of brain amyloidosis. Clin. Chim. Acta 2021, 519, 267–275. https://doi.org/10.1016/j.cca.2021.05.011.

Table S3: Analytical Validation Data and Specifications of the LC-MS/MS Assays Used in the PrecivityAD2 Blood Test [14,20,21]

**Analytical Performance Variables**	**Summary Results**
Limits of detection	Limit of detection is equal to the limit of quantitation; *Abeta40 LOQ*: 11 pg/mL; *Abeta42 LOQ*: 2 pg/mL; *np-tau217 LOQ*: 7 pg/mL; *p-tau217 LOQ*: 1.3 pg/mL
Total within-laboratory imprecision	*Abeta40*: 2.7% to 7.7%<br>*Abeta42*: 3.1% to 9.5%<br>*np-tau217*: 5.4% to 9.9%<br>*p-tau217*: 7.3% to 9.7%.
Repeatability (within-day imprecision)	*Abeta40*: 1.5% to 3.0%<br>*Abeta42*: 2.5% to 8.4%<br>*np-tau217*: 6.0% to 8.8%<br>*p-tau217*: 7.3% to 12.0%

Accuracy confirmed through spike-and-recovery experiments	*For plasma Ab40 and Abeta42*: Acceptable recovery at low (96–108%), medium (102–113%), and high (100–114%) concentrations; *For np-tau and p-tau217*: Acceptable recovery at low (98-105%), medium (92–104%), and high (102-109%) concentrations
Linearity	*Abeta40*: 10 to 1,780 pg/mL *Abeta42*: 2 to 254 pg/mL *np-tau217*: 3 to 280 pg/mL *p-tau217*: 0.1 to 81 pg/mL

References: [14] Meyer, M.R.; Kirmess, K.M.; Eastwood, S.; Wente - Roth, T.L.; Irvin, F.; Holubasch, M.S.; Venkatesh, V.; Fogelman, I.; Monane, M.; Hanna, L.; et al. Clinical validation of the PrecivityAD2 blood test: A mass spectrometry-based test with algorithm com-bining %p-tau217 and Aβ42/40 ratio to identify presence of brain amyloid. Alzheimer Dement. 2024, 20, 3179–3192. https://doi.org/10.1002/alz.13764. [20] Eastwood, S.M.; Meyer, M.R.; Kirmess, K.M.; Wente-Roth, T.L.; Irvin, F.; Holubasch, M.S.; Verghese, P.B.; West, T.; Braunstein, J.B.; Yarasheski, K.E.; et al. PrecivityAD2™ Blood Test: Analytical Validation of an LC-MS/MS Assay for Quantifying Plasma Phospho-tau217 and Non-Phospho-tau217 Peptide Concentrations That Are Used with Plasma Amyloid-β42/40 in a Multi-analyte Assay with Algorithmic Analysis for Detecting Brain Amyloid Pathology. Diagnostics 2024, 14, 1739. https://doi.org/10.3390/diagnostics14161739. [21] Kirmess, K.M.; Meyer, M.R.; Holubasch, M.S.; Knapik, S.S.; Hu, Y.; Jackson, E.N.; Harpstrite, S.E.; Verghese, P.B.; West, T.; Fogelman, I.; et al. The PrecivityAD™ test: Accurate and reliable LC-MS/MS assays for quantifying plasma amyloid beta 40 and 42 and apolipoprotein E proteotype for the assessment of brain amyloidosis. Clin. Chim. Acta 2021, 519, 267–275. https://doi.org/10.1016/j.cca.2021.05.011.
